# Supplementary material for: Changes in lung function and dyspnea perception in Colombian Covid-19 patients after a 12-week pulmonary rehabilitation program
Source: PLoS One. 2024 Nov 15;19(11):e0300826. doi: 10.1371/journal.pone.0300826 (PMC11567541; doi:10.1371/journal.pone.0300826)
Supplement: Attachment — Submitted filename: Response to Reviewers.pdf [file pone.0300826.s003.pdf]

Rebuttal letter

Carolina Zona-Rubio
Research professor
Universidad Manuela Beltrán, Bogotá, Colombia.

July 2024

Dear Editor,

We wish to respond to the reviewers of our research article entitled "Changes in Lung Function and Dyspnea Perception in Colombian COVID-19 Patients After a 12-Week Pulmonary Rehabilitation Program." Below, we address each point. It is important to clarify that these changes have been applied to the manuscript.

1. **Is the manuscript technically sound, and do the data support the conclusions?**
    - Coherence errors between sections have been corrected.
2. **Have the authors made all data underlying the findings in their manuscript fully available?**
    - All files containing the raw data have been uploaded as supplemental material in Excel format.
3. **This study involved rehabilitation programs that had positive effects on respiratory function after SARS-CoV-2 infection. Here are my comments: The inclusion criteria did not discriminate between the degree of COVID-19 infection, which could affect the degree of lung injury. If the patients had only mild disease, recovery could occur spontaneously without rehabilitation.**
    - Severity criteria for SARS-CoV-2 infection have been incorporated into the methodology to support the findings of the study pre- and post-pulmonary rehabilitation.
4. **The FEF 25-75% was not different between both groups. Why do the authors conclude that this might be associated with changes in the small airways?**
    - This point has been corrected.
5. **The study did not measure previous lung function before COVID-19 infection; therefore, how can you conclude that the cases that did not improve could be explained by underlying lung disorders?**
    - Comorbidities were included as variables in the analysis. ANCOVA analysis showed no correlation between age, height, weight, and lung function measures (FVC, FEV1, MRC). Nested ANOVA indicated that the presence or absence of comorbidities did not affect the differences observed in FVC and FEV1 between men and women. The text has been adjusted accordingly. Indeed, we do not have previous data on lung function before COVID-19 infection.
6. **It would be better to perform DLCO testing in addition.**
    - DLCO testing was not performed because the research institution lacked this diagnostic capability, due to the technological availability in the Colombian region where the research was conducted.
7. **Your study is sound and practical regarding ventilation effectiveness and complications, and it demystifies exclusion criteria based on comorbidities and demographics. Intensivists and pathologists in other countries also reported alveolar damage depending on the parameters. I noticed the issues below that, if addressed, will improve the quality of your paper: Sample size**

n=100 with N=231 corresponds to e=7.5% instead of 5%; either adjust CI or run the analysis with n=146.

- This point has been corrected.

8. **Normalize VMN/VMNI, IV/NIV, and IMV/NIMV abbreviations, as well as variables, e.g., mMRC/MRC.**
   - This point has been corrected.
9. **Missing decimal point in Height's p-value in Table 3.**
   - This point has been corrected.
10. **Two plots are named FEV1 in Figure 1.**
    - This point has been corrected.
11. **Figure 3 is difficult to read for individuals with color blindness.**
    - All figures have been reedited. We employed the IBM Color Blind Safe Palette as follows: Ultramarine 40, Indigo 50 (#785EF0), Magenta 50 (#DC267F), Orange 40 (#FE6100), Gold 20 (#FFB000).
12. **Add summary statistics for spirometry variables pre- and post-intervention (e.g., in Table 2).**
    - We uploaded a file with the summary statistics for spirometry variables pre- and post-intervention as supplemental material.
13. **Supporting the lack of obstructive patterns before PR (in Discussion).**
    - While we acknowledge the importance of expanding the study as suggested, it is not possible to do so due to financial limitations. This is a common challenge in Latin America. However, we consider the study findings to be relevant as they demonstrate the effects of pulmonary function before and after a rehabilitation program in a Latin American region.

Finally, we would like to request that the order of the authors presented in the manuscript be maintained. At the time of submitting the manuscript, the page did not allow selecting who is the corresponding author and who is the first author.

Please address all correspondence concerning this manuscript to me at dczonar@unal.edu.co. Thank you for your consideration of this manuscript.

Sincerely,

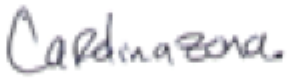

Carolina Zona
