# Supplementary material for: Changes in lung function and dyspnea perception in Colombian Covid-19 patients after a 12-week pulmonary rehabilitation program
Source: PLoS One. 2024 Nov 15;19(11):e0300826. doi: 10.1371/journal.pone.0300826 (PMC11567541; doi:10.1371/journal.pone.0300826)
Supplement: Attachment — Submitted filename: Response to Reviewers Sept 2024.pdf [file pone.0300826.s004.pdf]

Rebuttal letter

Carolina Zona-Rubio

Research professor

Universidad Manuela Beltrán, Bogotá, Colombia.

September 2024

Dear Editor's,

Thank you for the opportunity to revise our manuscript titled "Changes in Lung Function and Dyspnea Perception in Colombian COVID-19 Patients After a 12-Week Pulmonary Rehabilitation Program." We appreciate the valuable feedback from you and the reviewers, which has helped us enhance the quality of our work. Below, we address each point raised by the reviewers.

Abstract

1. **Use of full forms for PR and mMRC**: Thank you for your suggestion. We have revised the manuscript to include the full forms of PR (pulmonary rehabilitation) and mMRC (modified Medical Research Council) the first time they appear.
2. **Mentioning exact p-values**: We acknowledge that Multidimensional Scaling (MDS) and Linear Discriminant Analysis (LDA) do not provide p-values, as they are classification techniques rather than hypothesis-testing methods. However, we have included exact p-values for all relevant univariate analyses in the results section to enhance clarity.
3. **Limitation about sample size in the abstract conclusion**: We have removed the statement regarding the need for a larger sample size from the conclusion of the abstract, recognizing it as a limitation more suitable for the discussion section.

Introduction

1. **Ethical guidelines statement**: We have moved the sentence regarding the Ethical Guidelines for Human Research from the Declaration of Helsinki from the introduction to the methods section, as suggested.
2. **Omitting unnecessary sentences**: We have shortened the introduction by removing the following sentences:

- "Which involves evaluating the mechanical properties of the respiratory system through volumes and capacities; it provides relevant information for the classification of both obstructive and restrictive disorders."
- "Which has motivated evaluations of lung function to characterize changes in the lung parenchyma and assess the need for treatment."

Materials and Methods

1. **Use of FEF and PEF in full**: We have made the requested adjustments and now use FEF (forced expiratory flow) and PEF (peak expiratory flow) in full the first time they appear.
2. **Intensity of exercises**: The intensity of exercises has been specified in the revised manuscript.
3. **Warm-up and cool-down periods**: We have rectified the oversight regarding warm-up and cool-down periods, ensuring that training sessions now include both.
4. **Reducing use of "we"**: We have thoroughly revised the manuscript to reduce the frequency of the word "we" and adopted a more passive voice, making the writing more objective.
5. **Reference for symptom severity classification**: We have added the appropriate reference to support the classification of symptom severity.

Discussion

1. **Clarifying limitations**: We have clarified the limitations of our study at the end of the discussion section, as suggested.

Attached, we are sending the unmarked version of the document, as well as the version with track changes to facilitate the review and reading of the changes. We believe these revisions address all the concerns raised by the reviewers.

Thank you for your consideration of our manuscript. We look forward to your positive response.

Sincerely,

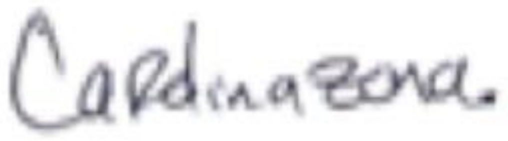

Carolina Zona
